# Relationship of ultraviolet light exposure and cutaneous and systemic disease activity in youth with childhood-onset systemic lupus: Results from the Childhood Arthritis and Rheumatology Research Alliance Registry

**DOI:** 10.21203/rs.3.rs-3777774/v1

**Published:** 2024-01-03

**Authors:** Tamara I. Tanner, Ilir Agalliu, Dawn M. Wahezi, Tamar Rubinstein, CARRA Registry Investigators

**Affiliations:** Albert Einstein College of Medicine; Albert Einstein College of Medicine; Albert Einstein College of Medicine; Children’s Hospital at Montefiore; Childhood Arthritis and Rheumatology Research Alliance

**Keywords:** Lupus, Pediatric, Childhood-onset lupus, Cutaneous lupus, Ultraviolet, Environmental exposure

## Abstract

**Objective:**

To investigate the association between sun exposure measured by ultraviolet light index (UVI) and seasonality with rash and systemic disease activity in youth with childhood-onset systemic lupus (cSLE) from the Childhood Arthritis and Rheumatology Research Alliance (CARRA) Registry.

**Methods:**

We reviewed data on rash and disease activity from Systemic Lupus Erythematosus Disease Activity Index 2000 (SLEDAI-2K) scores from cSLE CARRA Registry participants with visits between 2010 and 2019 and obtained UVI data from the National Oceanic and Atmospheric Administration (NOAA). Our main exposures were UVI and season during the month of visit and one month prior to visit. We used mixed-effects logistic regression to examine an association between UVI/season and rash / SLEDAI-2K score, adjusting for age, sex, race and income.

**Results:**

Among 1222 participants, with a mean of 2.3 visits/participant, 437 visits (15%) had rash and 860 (30%) had SLEDAI-2K score ≥ 5. There were no associations between UVI during the month prior to visit, or the month of the visit and odds of rash or elevated systemic activity. However, fall season was associated with increased odds of rash (OR = 1.59, p = 0.04), but there not increased disease activity.

**Conclusion:**

While we found no association between UVI and rash or UVI and disease activity, further studies directly measuring UVI may help further understand whether a relationship exists between sun exposure and SLE disease activity and whether this is an area that continues to require clinical attention.

## INTRODUCTION

Environmental factors, including sunlight and UV radiation, have been identified as important contributors in the pathogenesis of several autoimmune diseases, among them systemic lupus erythematosus (SLE) [1]. Ultraviolet radiation (UVR) is thought to be a trigger for SLE flares and in particular flares of cutaneous disease or rash. Photosensitive rash is one of the original diagnostic criteria for SLE [2] and skin lesions are found in more than 50% of patients with SLE [3]. Limiting sun exposure is potentially one of the few modifiable risk factors for lupus disease activity, and as such, improving our understanding of this ubiquitous risk factor is of utmost importance.

Seasonal variations in SLE disease activity have been described [4–6], however there is conflicting evidence as to whether an association between sun exposure and disease activity exists, even in regard to cutaneous manifestations. In addition, few studies have examined ultraviolet index (UVI) against season to determine whether previously described variations in disease activity are really due to UV exposure versus other possible factors. While most studies that have examined associations between UVR and SLE have been conducted in adults, rash and photosensitivity may be even more prevalent in youth with childhood-onset SLE (cSLE) [7]. Furthermore, even though skin pigmentation alters the risk for UVR-related damage, only a few studies have included diverse populations, demonstrating variation in the prevalence of rash across different ethnic origins, with increased photosensitivity prevalence in people of European ancestry as opposed to people of African ancestry [8–10].

The aim of this study was to assess the association between UV exposure (quantified by UVI) and seasonality with risks of cutaneous and systemic disease activity in a cohort of racially and ethnically diverse youth with childhood-onset lupus enrolled in the Childhood Arthritis and Rheumatology Research Alliance (CARRA) Registry. Based on prior studies, we hypothesized that increased disease activity, especially cutaneous flares, would be associated with elevated UVI.

## METHODS

### Participants

Study participants included all youth aged 0 to 21 years of age with SLE who were enrolled in the Childhood Arthritis and Rheumatology Research Alliance (CARRA) Legacy and New (current) Registry databases. The CARRA SLE Registries comprise data on youth with childhood-onset lupus diagnosed by a pediatric rheumatologist before their 18th birthday and enrolled before the age of 21.

The CARRA Legacy Registry enrolled 1,284 participants with SLE between 2010 and 2015 from 65 participating sites (64 in the US and one in Canada); while the CARRA New Registry enrolled 514 participants with SLE from June 2016 through June 2019 (time of our data request) from 77 participating sites, mostly in the US and Canada. For this analysis, we included only participants residing in the US and with a valid US zip code due to the availability of reliable UVI data, which we obtained from the National Oceanic and Atmospheric Administration (NOAA).

Information obtained from the CARRA Registry database included date of visit, 5-digit zip code at time of visit (to be able to cross reference with UV index database obtained from NOAA). Demographic data was entered by participants or parents including sex, age, race and ethnicity, household income, education (New Registry only). Total and component Systemic Lupus Erythematosus Disease Activity Index 2000 (SLEDAI 2K) scores were entered by research staff based on clinical visits. Exclusion criteria include incomplete data collection for the variables of participant zip code and date (month, year) of clinic visit, incomplete clinical data such as presence of rash or SLEDAI score.

The study was approved by the Einstein/Montefiore IRB 2018-9819.

### Data source

Ultraviolet Index (UVI) data for the month and year of visits for each participant was obtained from NOAA for specific zip codes of each patients’ visits. The UVI represents the estimated amount of skin-damaging UV radiation reaching the earth’s surface at solar noon and is an internationally standardized unit on a linear scale, ranging from 0 to the 11+ [11]. A higher number indicates a shorter amount of time to skin erythema. UV calculation considers other elements such as latitude, total column ozone, elevation, earth surface characteristics, cloud cover, and tropospheric aerosol loading (pollutants or dust).

### Exposures

The primary exposure was UVI of the month and year of the participant’s visit as well as month prior to visit, to account for latency (lag) between exposure and onset of symptoms [12]. We also used seasonality (i.e. spring, summer, fall and winter) as a secondary measure of exposure and examined the association of season with rash and elevated systemic disease activity. For this analysis we used the meteorological definition of season, which defines seasons as beginning at the first day of the month that includes the equinoxes and solstices; for example according to this definition summer begins June 1 and ends August 31 [13].

### Outcomes

Rash: The primary outcome was presence of inflammatory rash at the time of the visit defined by the SLEDAI-2K or SLEDAI rash component. SLEDAI is a global index that evaluates disease activity over the previous 10 days. It includes 24 items with specific manifestations in 9 organ systems: neurological, vascular, musculoskeletal, renal, serosal, mucocutaneous, constitutional, immunological and hematological[14]. The SLEDAI rash score is defined as positive if an SLE-related inflammatory rash is present from up to 10 days prior to the time of visit. On the SLEDAI-2K (used in the CARRA Registry) this includes new and persistent rash [15].

Elevated systemic disease activity: Definition of active disease in SLE is not standardized but using SLEDAI activity measure is usually defined as increase in score by > 3 for mild/moderate flare and > 10 for severe activity [16]. Since this was difficult to assess using our database, we used SELDAI score for each visit to assess for disease activity. We used total SLEDAI score ≥ 5 to define elevated systemic disease activity, as studies have defined scores < 5 as low disease activity state [17]. Thus, our secondary outcome was elevated systemic disease activity measured as SLEDAI score score ≥ 5.

**Figure 1:**
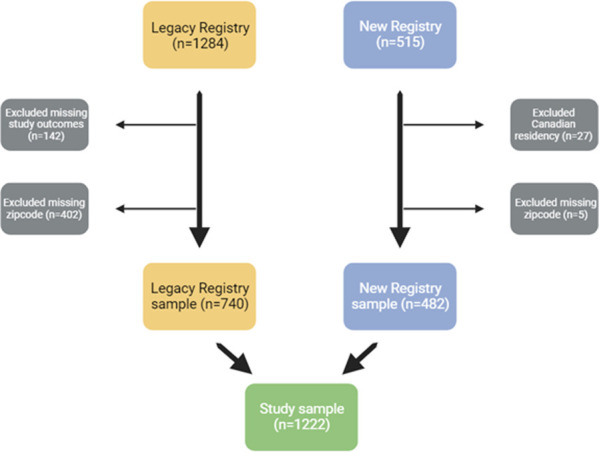
Flowchart of study sample from CARRA Registry cSLE participants (legacy and new)

We excluded patients that were missing zip code information and/or had Canadian residency (n = 174) or those who had missing/unknown information on the outcomes of interest (n = 402) (Fig. 1).

### Statistical analysis

We compared socio-demographic characteristics between the two registries using Student T-tests for normally distributed variables and chi-square tests for categorical variables. Statistical significance was determined as p < 0.05 (2-sided). We used mixed-effects logistic regression to examine association between UVI (both as continuous and categorical) and meteorological definition of season (i.e. spring, summer, fall vs. winter) with risks of rash (yes vs no) as well as SLEDAI score (using ≥ 5 vs 0–4) in separate logistic regression models. Our main UV exposures were UVI index of the month/year of participants’ visits as well as UVI index of the month prior to visit date. We used the meteorological cut-off point for UVI index to indicate, low, medium high and very-high UV exposure [11]. These models use person as random effect to account for repeated measures within the same subject from baseline and follow-up visits and we adjusted for potential confounding by sex, age, race/ethnicity and household income as fixed effects. All analyses were conducted using STATA software version 16.

## RESULTS

### Participant demographics and clinical characteristics

We examined all available visits (baseline and follow-up) for each Registry participant. We included 740 Legacy Registry participants, accounting for 1703 visits and 482 New Registry participants with a total of 1128 visits for an overall total of 2831 visits. Demographics of participants in the Legacy Registry and the New Registry are shown in Table 1. Overall, most participants were female (84%), 30% identified as Black and 25% as Hispanic, and approximately a third of participants had low household income <$50,000 (394, 32%).

Participants from Legacy Registry were slightly older at baseline (average age 15.8 ± 3.2) compared to the New Registry (average age 14.4 ± 2.9), but there was no meaningful difference in sex, race/ethnicity, or family income distribution. Information about educational attainment was collected only in the New Registry database (Table 1). Overall, there were 2,831 visits among 1,222 participants (average 2.3 visits / patients), and the frequency of visits was similar in both registries.

### Rash

Among all visits included in the analysis, 437 (15%) had reported a rash event (Legacy Registry 249, 15%; New Registry 188, 17%). Table 2 shows associations between UVI index and seasonality and odds of rash. There was no association between UV index of the month (OR = 0.96 CI 0.92–1.03) as well as UV prior to visit or the month of the visit (OR = 0.99, CI 0.93–1.05) and rash. Similarly we didn’t see any association when UV index was categorized according to level (low 1–2, moderate 3–5, high 6–7, very high 8–10) [11]. When looking at seasons, fall season was associated with increased odds of rash (OR = 1.61, CI 1.03–2.52, p = 0.04) however there was no association when looking at the visit months.

### Elevated Systemic Disease Activity

Among all visits included in the analysis, 860 (30%) had SLEDAI ≥ 5, indicating elevated systemic disease activity; interestingly the proportion of patients reporting a SLEDAI ≥ 5 was lower in Legacy Registry (473, 27%) compared to New Registry (387, 35%).

**Figure 2:**
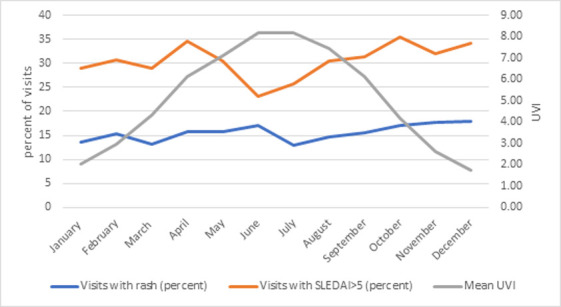
Seasonal distribution of systemic lupus disease activity and rash in CARRA Registry cSLE participants

Unlike some prior studies which showed increased seasonal disease activity [18–20], our study did not find an association between UV exposure or season and active disease (Table 3). When looking at UV exposure both during current and previous month, there were no increased odds of active disease (OR = 0.99 CI 0.94–1.04); this remained true when we stratified by UV levels, or when we looked at seasons. Figure 2 shows the monthly distribution of visits with rash and elevated systemic disease activity when compared with mean monthly UVI.

## DISCUSSION

In this examination of youth with cSLE enrolled in the CARRA Registry, we found that fall season was associated with 30% increased odds of rash when compared to winter. We otherwise found no significant association between UV exposure and cutaneous or systemic disease activity; this was true when analyzing the relationship between UVI of the month prior to each visit or the month of each visit, and remained true when adjusting for age, sex, race/ethnicity and income.

Photosensitivity has long been implicated as one of the environmental risk factors for many autoimmune diseases, and SLE is one of the most described photosensitive autoimmune disease. Photosensitivity was included as one of original ACR diagnostic criteria for SLE, although this criterion proved to be controversial, as being poorly defined and overlapping with other criteria such as malar rash and thus was not included in the most recent 2019 EULAR/ACR classification criteria [21].

Studies have shown multiple mechanisms by which solar irradiation causes tissue damage. These include free radical formation, deregulated apoptosis, activation of plasmacytoid dendritic cells which secrete INF-alpha, upregulation of TNF-alpha and other pro-inflammatory cytokines, all of which have been implicated in SLE pathogenesis [22]. However, epidemiological studies looking at patients with different forms of Lupus Erythematosus (LE) report a wide range of photosensitivity rates (from 27–100% for SCLE, 25–90% for discoid lupus, and 43–71% for lupus tumidus) [8], which likely reflect the difficulty in defining and assessing photosensitivity. Studies have shown that patient history corelates poorly with presence of photosensitivity [8], possibly due to the delayed nature of the process. The delay between exposure to UV radiation and onset of rash has been shown in phototesting studies, with studies showing that up to 90% of patients with LE had abnormal reaction to phototesting, however the delay between testing and onset of rash was in the order of days to weeks [23].

It is important to note, however, that phototesting does not correlate well with patients’ and physicians’ subjective assessments of photosensitivity, interjecting doubt as to whether phototesting studies reliably reflect clinical disease flare [12].

When looking at seasonal influence as a possible measure of UV radiation on SLE disease activity the results are conflicting, with some studies showing increased evidence of rashes in summer months [5, 6, 18] while other studies did not show this relationship [4, 19]. A large prospective longitudinal cohort study [5] showed an increase in both photosensitive rash and arthritis activity in the spring and summer months (interestingly, rash only had seasonal variation in white but not African American participants). For systemic disease the evidence is also mixed, with Hopkins Lupus Cohort study [5] showing decreased of renal flares in the summer and highest anti-DsDNA levels in winter, while other studies [19, 20] showed higher lupus flare rates in winter months.

Studies have also looked at the relationship between UV radiation and season in other autoimmune diseases. A recent study of youth with Juvenile Dermatomyositis enrolled in the CARRA Registry found no significant association between mean UVI and calcinosis and other measures of disease severity. However, the odds of calcinosis markedly decreased in African American participants and steadily increased in non–African American participants over the range of increasing mean UVI [24]. The seasonal rates of flare in other autoimmune disease have also been described, especially multiple sclerosis, psoriasis, rheumatoid arthritis [25].

The effect of UV radiation on autoimmune disease is not straightforward; while UV (especially UV-B) has been implicated in pathogenesis of photosensitivity, the immunomodulatory beneficial role of UV-A (especially UV-A1) phototherapy has also been described [26]. Another related factor that has been proposed to have a role in SLE pathogenesis is vitamin D, the production of which is dependent on UV; several studies have found that higher vitamin D levels were associated with lower disease activity scores [27]. In addition, studies of seasonal characteristics of disease must take into account other factors associated with seasonality such as melatonin levels (which have been shown to have an immunomodulatory effect and are lowest in spring), and the role of infections (which are usually more prevalent in cold months). Different studies address some of these factors but not in a consistent way.

The vigilant use of regular sun protection is a common consensus recommendation in most published guidelines on SLE care [28, 29] despite lack of clear evidence. Our study did not find a clear association between UV exposure and rash or increased disease activity, although this is clearly an area that warrants further study. Multiple studies have emphasized the role of environmental factors in disease onset and activity, and UV radiation is the single most important risk factor identified in in-vitro and clinical studies.

Our study has several limitations. First, the CARRA Registry collects data every 6 months which means disease flares may be missed if they occur between the 6-months data collection visits. Unscheduled visits are included in the Registry at times of changes in medication, which can potentially address this issue partially, however medication changes might be due to flare or medication intolerance; thus not all disease flares are captured in the registry which is an important limitation. We used UVI to infer sun exposure, which is an indirect marker; thus, we are lacking information on factors such as individual behavior, extent of sun exposure/time spent outdoors, use of sun protective measures such as sunscreen and clothing, levels of vitamin D and presence of infection. We also used the SLEDAI category of rash as our primary outcome; while the SLEDAI definition is presence of ‘inflammatory rash’ it is not clear that everyone defines inflammatory rash in similar ways, which is potentially a limitation. In addition, we used UVI to estimate UV radiation exposure, while some investigators argue that UV-B flux, a composite measure of mean UV-B radiation level based on latitude, altitude and cloud cover, better represents ambient exposure [8]. It is important to note that while we adjusted for race/ethnicity in our analyses, this variable represents a social construct that relates to risk associated with racism and structural barriers, not genetic ancestry or phototype. Further studies with characterized phototype may reveal that the relationship between UVR and rash or disease activity may differ across differently melanated skin; this could not be examined in this study with the available Registry data.

Despite these limitations, this is the largest study to date and first of its kind to evaluate the association between UVR with both rash and systemic disease activity in cSLE. This study leverages the rich longitudinal database of cSLE participants available in the CARRA Registry as well as a NASA database of daily recorded UVI values; patients enrolled in the registry are from a diverse geographical distribution which helped provided a more thorough picture for sun exposure.

## CONCLUSION

Though sun protection is emphasized in SLE care, the relationship between UV and SLE disease, even specifically cutaneous disease, remains unclear. Further studies that more directly measure UVI may be warranted to better understand this relationship and whether this is an area that continues to require our attention for prevention of SLE disease activity.

## Figures and Tables

**Table 1:** Demographic and social characteristics of all participants included in this analysis as well as stratified by registry

	All participants (n = 1222)	Legacy Registry n = 740	New Registry n = 482
Age at visit (years, mean, SD)	15.2 (3.2)	15.8 (3.2)	14.4 (2.9)
Sex, n(%)			
Female	1023 (83.7)	619 (83.6)	404 (83.8)
Race/Ethnicity; n(%)			
Non-Hispanic White	387 (31.7)	248 (33.5)	139 (28.8)
Non-Hispanic Black	361 (29.5)	224 (30.3)	137 (28.4)
Hispanic	310 (25.4)	181 (24.5)	129 (26.8)
Other/unknown	164 (13.4)	87 (11.8)	77 (16)
Income			
<25k	194 (15.9)	131 (17.7)	63 (13.1)
25–49.9k	200 (16.4)	126 (17)	74 (15.4)
50–74.9k	118 (9.7)	75 (10.1)	43 (8.9)
75–99.9k	102 (8.4)	61 (8.2)	41 (8.5)
100–150k	112 (9.2)	71 (9.6)	41 (8.5)
>150k	91 (7.5)	47 (6.4)	44 (9.1)
Unknown and missing	405 (33.1)	229 (31)	176 (36.5)
Education; n(%)			
Elementary/middle school		--	17 (3.8)
Some high school		--	23 (5.2)
Graduated high school		--	103 (23)
College		--	163 (36.5)
Graduate school		--	63 (14.1)
Prefer not to answer		--	78 (17.5)
Total number of visits (baseline and follow-up)	2831	1703	1128
Visits with rash	437 (15%)	249 (15%)	188 (17%)
Visits with SLEDAI ≥ 5	860 (30%)	473 (27%)	387 (35%)

* Data not available in the Legacy Registry

**Table 2:** Odds of rash by ultraviolet index (UVI) and seasonality among the youth with cSLE from CARRA Registries

	Crude			Multivariate-Adjusted		
	OR	95% CI	P value	OR	95% CI	P value
UVI (continuous)	0.97	0.92–1.03	0.39	0.97	0.92–1.04	0.42
UV lag (continuous)	0.99	0.93–1.05	0.65	0.99	0.93–1.05	0.70
UVI category Low	1	ref	--	1	ref	--
Moderate	0.78	0.52–1.18	0.24	0.78	0.52–1.18	0.24
High	0.67	0.44–1.01	0.06	0.66	0.44–1.01	0.06
Very high	1.08	0.65–1.8	0.78	1.08	0.65–1.81	0.76
Season						
Winter	1	ref	--	1	ref	--
Spring	1.22	0.8–1.87	0.37	1.23	0.8–1.89	0.35
Summer	1.00	0.65–1.55	0.99	1.02	0.65–1.58	0.94
Fall	**1.59**	**1.02–2.47**	**0.04**	**1.61**	**1.03–2.52**	**0.04**

* Adjusted for age, sex, race and income

* UVI Ultra Violet Index

* UVI, UVI lag, UVI categories and seasons were run as separate models

* Seasons are defined as follows: winter January 1-March 31, spring April 1-June 30, summer July 1- September 30, fall October 1-December 31

* UVI categories: low 1–2, moderate 3–5, high 6–7, very high 8–10[11]

**Table 3:** Odds of elevated systemic lupus disease activity (SLEDAI ≥ 5) by UVI and season in youth with cSLE from the CARRA Registry

	Crude			Multivariate-Adjusted*		
	OR	95% CI	P value	OR	95% CI	P value
UVI***	0.99	0.94–1.04	0.64	0.99	0.94–1.04	0.63
UV lag	0.99	0.94–1.03	0.55	0.98	0.94–1.03	0.52
UVI cat*** Low	1	ref	--	1	ref	--
Moderate	1.02	0.74–1.4	0.91	1.0	0.73–1.38	0.97
High	0.95	0.69–1.3	0.73	0.95	0.69–1.31	0.76
Very high	0.81	0.55–1.21	0.31	0.81	0.54–1.21	0.30
Season^						
Winter	1	ref	-	1	ref	--
Spring	1.01	0.73–1.40	0.93	1.02	0.73–1.41	0.92
Summer	1.02	0.74–1.41	0.90	1.05	0.76–1.46	0.76
Fall	1.29	0.92–1.81	0.14	1.29	0.92–1.81	0.14

* adjusted for age, sex, race, and income

** UVI: Ultra Violet Index; UVI, UVI lag, UVI categories, and seasons were all analyzed as separate models.

*** UVI categories: low 1–2, moderate 3–5, high 6–7, very high 8–10[11]

^ seasons defined as: winter January 1-March 31, spring April 1-June 30, summer July 1-September 30, fall October 1-December 31

## Data Availability

Data sources are available through the Childhood Arthritis and Rheumatology Research Alliance Registry (CARRA) and NOAA (National Oceanic and Atmospheric Administration). CARRA Registry data used in this study was made available to the authors through a data use agreement. Data from the CARRA Registry is not publicly available, but available from CARRA by request (carragroup.org)

## Abbreviations

ACR: American College of Rheumatology
CARRA: Childhood Arthritis and Rheumatology Research Alliance
cSLE: Childhood-onset systemic lupus erythematosus
DsDNA: Double-stranded deoxyribonucleic acids
EULAR: European Alliance of Associations for Rheumatology
INF: Interferon
NASA: National Aviation and Space Administration
NOAA: National Oceanic and Atmospheric Administration
SLE: Systemic lupus erythematosus
SLEDAI: Systemic Lupus Erythematosus Disease Activity Index
TNF: Tumor necrosis factor
UV: Ultraviolet
UVI: Ultraviolet index
UVR: Ultraviolet radiation
